# Educating and learning in the Artificial Intelligence era: formative challenges and new roles for the educator

**DOI:** 10.31744/einstein_journal/2026Suppl_1ED0001

**Published:** 2026-02-05

**Authors:** Dannielle Fernandes Godoi, Andrea Gomes da Costa Mohallem, Blaidi Sant´Anna, Elda Maria Stafuzza Gonçalves Pires, Fernanda Domingos Giglio Petreche, Luciana Machado Paschoal, Thomaz Bittencourt Couto, Mariana Lucas da Rocha Cunha

**Affiliations:** 1 Hospital Israelita Albert Einstein São Paulo SP Brazil Hospital Israelita Albert Einstein, São Paulo, SP, Brazil.

## What is lost when generating answers becomes more important than learning?

Artificial intelligence (AI) has ceased to be a prospect and has become a routine tool for study, academic production, and decision-making. Over the past three years, language models based on generative AI have migrated from an interesting technological curiosity to widely available tools, with unprecedented speed and impact at scale across multiple fields, including education. And it is precisely in education that this movement has revealed an inflection point still underway: students have incorporated AI into their academic routines before institutions and educators have been able to agree on principles, limits, and formative objectives for its use.

The challenge, however, is not technological in its essence; it is pedagogical. Teaching and learning are deeply contextual, human, and relational processes, permeated by multiple variables, including the methodologies employed, individual cognitive difficulties, life and sociocultural contexts, and even fragmented attention, which is common among the current generation. These factors are not resolved by faster access to answers. The temptation to use AI as a "shortcut," something we have frequently observed in different educational environments, results in rapid knowledge acquisition at the expense of deep and effective learning. Immediate answers reduce the effort required to build understanding, judgment, and autonomy for decision-making in the real world.

The scale of adoption underscores the urgency of this discussion across different educational settings. International multicenter studies indicate that 86% of higher education students already use artificial intelligence tools in their studies, with more than half doing so at least weekly and nearly a quarter daily.^(1)^ In the same context, more than 60% of educators report having already used AI in teaching activities, although the majority express significant concerns regarding students’ excessive dependence and their ability to critically evaluate content generated by algorithms.^(2)^ In Brazil, recent data point not only to the widespread use of AI in higher education,^(3)^ but also to its growing penetration in primary and secondary education,^(4)^ albeit unevenly across educational contexts.

These figures reveal a critical mismatch: while the use of AI becomes normalized in students’ daily lives, institutional and pedagogical responses are still under development. In this context, the absence of qualified mediation by educators does not interrupt students’ use of AI, but rather redefines, in a concerning way, how learning occurs, and which skills and competencies fail to be developed in this process. This is not, therefore, a discussion restricted to technology-related courses, but rather an examination of a transversal shift in the educational ecosystem: AI has entered unannounced and is unlikely to leave.

It is important to understand this rapidly disseminated use of AI through a broader perspective on its possible causes. For many students, AI offers more accessible language, immediate feedback, reduced insecurity, and time savings. At the same time, in many educational environments, we still find rigid processes, teaching methodologies already proscribed by the scientific evidence, and the role of the student as the center of the educational process remains far below what it should be. Educational systems are historically slow and do not tend to be responsive to societal needs in real time. And when these same systems are pressured by time constraints, content-based assessment, and performance demands, the tool that "delivers" quickly tends to become the standard.^(5)^

Emerging scientific evidence points to an ambivalent picture. The use of AI may increase perceived efficiency, but it may also reduce direct cognitive engagement, with potential impacts on skills such as analysis, synthesis, and long-term retention. Recent studies describe effects ranging from overconfidence, characterized by the overestimation of one's own competence when performing AI-mediated tasks to users who stop reflecting on their tasks when assisted by AI.^(6-8)^ This combination (less effort, more confidence) is particularly sensitive in health education, where learning involves judgment under uncertainty, integration of incomplete information, and ethical responsibility in decisions that affect real people.^(9,10)^ At the same time, another body of evidence shows that AI can improve learning when it acts as a "cognitive tutor," that is, when students engage in dialogue, ask for explanations, contrast alternatives, and make their reasoning explicit.^(11-13)^ In other words, the risk is not the tool itself, but rather how it is used that defines the type of learning it encourages.

**Figure 1:**
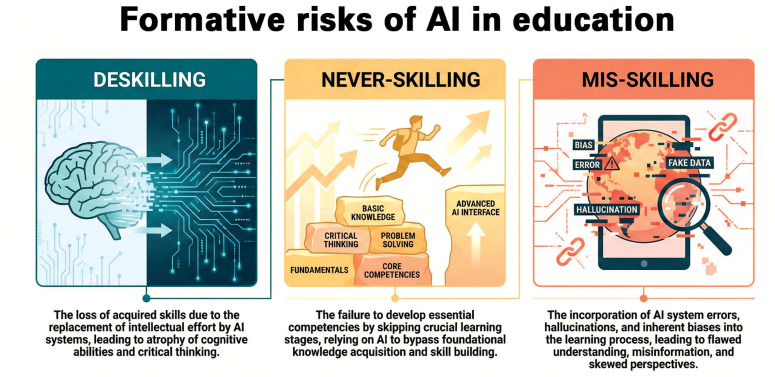
Learning risks associated with a lack of pedagogical mediation amidst the adoption of technologies

Source: Elaborated with Gemini based on data from Abdulnour et al.^(10)^

In this scenario, it becomes increasingly clear that when pedagogical mediation does not keep pace with this technological adoption, specific formative risks emerge, and it is important to conceptualize them to organize this debate (Figure 1). The first is deskilling: the loss of previously acquired skills due to the recurrent replacement of intellectual effort by AI. The second is never-skilling: the failure to develop essential competencies because fundamental stages of learning are no longer experienced. The third is mis-skilling: the incorporation of system errors and biases into the learning process. These three phenomena describe formative trajectories that may produce a substantial impact on an entire generation of new professionals at a time when educational institutions are not sufficiently prepared for this disruptive transformation of education.^(10)^

Understanding these three major risks and creating spaces for discussion about them is essential. The incorporation of new technologies into education has never been instantaneous. As occurred with active learning methodologies, simulation, and competency-based assessment, there is an inevitable learning curve for educators. Avoiding exposure to AI in educational processes may preserve familiar practices in the short term, but it increases the risk of disconnection between what happens in the classroom and how students construct knowledge outside of it.

On the other hand, a more restrictive adoption of artificial intelligence by educators may negatively impact the evolution of pedagogical practices and, consequently, contribute to training that is misaligned with the demands of students and the world of work. The reasons for this limitation are multifaceted and include both practical concerns related to the use of innovation, such as usability, pedagogical value, and perceived risks, and barriers of a psychological and cultural nature, involving educators’ beliefs, perceptions, and prior experiences.^(14,15)^ Institutional policies should address these concerns through strategies for training, continuous support, and clear guidance for the pedagogical use of AI. In addition, it is essential to foster an ongoing academic debate that allows for balancing the potential gains of the technology with its ethical challenges, ensuring that its incorporation strengthens (rather than weakens) human creativity, critical thinking, and professional teaching identity.

The educational response, therefore, is not about resisting AI as if it were possible to "go back in time," but about repositioning the entire educational process. The debate matures when it moves away from "allowing or not allowing" and enters the realm of evidence and institutional responsibility. Prohibiting the use of AI without institutions and educators preparing themselves to understand it, experiment with it, and critically integrate it into educational processes does not eliminate the problem; it merely shifts it outside the pedagogical space. When use remains invisible, the opportunity to guide, regulate, and transform the tool into an explicit object of learning is lost.

For artificial intelligence to truly become a powerful (and safe) tool in the educational field, deliberate investment will be required in instructional design, assessment aligned with complex cognitive processes, faculty development, and critical AI literacy, both among educators and students. This movement is central to preserving the quality of education in a context of rapid technological transformations and does not end with AI. And that is precisely what is at stake: not only how we use AI, but what we fail to learn when the answer comes before thought.

## Data Availability

The content is already available.
